# Thermal and Mechanical Variation Analysis on Multi-Layer Thin Wall during Continuous Laser Deposition, Continuous Pulsed Laser Deposition, and Interval Pulsed Laser Deposition

**DOI:** 10.3390/ma15155157

**Published:** 2022-07-25

**Authors:** Liang Ma, Xiangwei Kong, Jingjing Liang, Jinguo Li, Cong Sun, Zhibo Jin, Zhidong Jiao

**Affiliations:** 1 School of Mechanical Engineering and Automation, Northeastern University, Shenyang 110819, China; maliangneu@163.com (L.M.); suncong1@mail.neu.edu.cn (C.S.); zhibojin@163.com (Z.J.); 2 Institute of Metal Research, Chinese Academy of Sciences, Shenyang 110016, China; 3 CRRC Qingdao Sifang Co., Ltd., Qingdao 266000, China; a1162244303@163.com

**Keywords:** direct laser deposition (DLD), pulsed laser, finite element model, heat transfer, residual stress

## Abstract

Direct laser deposition (DLD) is widely used in precision manufacturing, but the process parameters (e.g., laser power, scanning patterns) easily lead to changes in dimensional accuracy and structural properties. Many methods have been proposed to analyze the principle of distortion and residual stress generation, but it is difficult to evaluate the involvement of temperature and stress in the process of rapid melting and solidification. In this paper, a three-dimensional finite element model is established based on thermal–mechanical relationships in multilayer DLD. Differences in temperature and residual stress between continuous laser deposition (CLD) and pulsed laser deposition (PLD) are compared with the numerical model. To validate the relationship, the temperature and residual stress values obtained by numerical simulation are compared with the values obtained by the HIOKI-LR8431 temperature logger and the Pulstec μ-X360s X-ray diffraction (XRD) instrument. The results indicate that the temperature and residual stress of the deposition part can be evaluated by the proposed simulation model. The proposed PLD process, which includes continuous pulsed laser deposition (CPLD) and interval pulsed laser deposition (IPLD), were found more effective to improve the homogeneity of temperature and residual stress than the CLD process. This study is expected to be useful in the distortion control and microstructure consistency of multilayer deposited parts.

## 1. Introduction

As a type of effective metal additive in manufacturing methods with high reliability and applicability, direct laser deposition (DLD) is used as a conventional process for manufacturing under complex conditions, such as emergency maintenance and outer-space operations [1]. The DLD process becomes more significant in the manufacturing and repairing of high-value parts, complex parts, and multi-material composite parts [2]. The laser beam is used as an input energy to melt the metal powder. A powder nozzle is moved to a specified position by a preset program. The input material and energy can be accurately transported to the specific location. The concentration of energy input is higher than that of the ordinary welding process. The powder utilization rate of DLD is higher than that of selective laser melting (SLM) [3].

Many studies on DLD have been reported in recent years. The melting process of materials in DLD is transient. However, it is difficult to directly measure its temperature, stress, and other key parameters because of the instrument’s application limitations and complex thermal convection environment. The numerical model turns out to be an effective method for DLD research.

Temperature field is an important factor in the mechanical properties and microstructure of DLD parts. Various material melting and heat conduction models have been developed to predict the temperature of the deposited part. Gao et al. [4] analyzed the temperature field of a single-track deposition model with a calculated profile, and claimed that a more accurate track profile and maximum temperature was obtained. Kovalev et al. [5] proposed a numerical model that considers gas-dispersed flow phenomena in the laser cladding. Arrizubieta et al. [6] described the accuracy of the clad geometry and temperature with fluid-dynamic phenomena under different process parameters. These articles all claimed that precise geometry and temperature were obtained with computational fluid dynamics. However, the computational efficiency was still a factor to be considered compared with the finite element method. Sawant et al. [7] presented a finite element model to predict the dilution of single-track deposition in different process parameters. Zhan et al. [8] and Xie et al. [9] predicted the temperature of single-track deposition and analyzed microstructure evolution with the CA-FE model. Du et al. [10] researched the temperature gradient and solidification growth rate in molten pools, and they also studied the influence of solidification microstructure on these factors. Chen et al. [11] and Yan et al. [12] studied the effects of laser energy distribution on the temperature field and microstructure through the finite element simulation. Walker et al. [13] presented a Gaussian process regression model to predict the track profile, temperature fields, and stress fields. These articles all established single-track finite element models to obtain temperature fields and stress fields. However, the temperature field and stress field distributions of multi-layer models have significant influences on the deposition of DLD parts. Nikam et al. [14] presented a multi-layer finite element model to study the influence of deposition direction on temperature fields. Liu et al. [15] presented a finite element model to study the effect of dynamic preheating on the temperature gradient and cooling rate. Zhang et al. [16] established a cellular automation-finite element model to simulate the temperature fields in multi-layer deposition, and predicted the grain morphology with the solidification thermal history. Bailey et al. [17] established a finite element model for multi-track and multi-layer deposition to predict phase transformation kinetics and residual stresses.

Pulsed laser deposition samples were validated with better stress rupture properties [18] and controlled porosity properties [19]. Fewer articles have used numerical models to study temperature and stress fields in the pulsed laser deposition process. Li et al. [20] established a single-track melt pool transport model to compare the temperature fields and dendrite growth between the pulsed wave deposition and continuous wave deposition. Han et al. [21] established a single-track numerical model to obtain the temperature, velocity, and stress fields in pulsed laser deposition. Ci et al. [22] predicted the primary dendritic arm spacing in a pulsed laser remelting process with a single-spot heating model.

Since the DLD process and pulsed laser are applied to multi-layer and multi-track additive manufacturing at the part level, high-efficiency and low computational cost models based on thermophysical parameters have become a new research focus.

To fill this gap, a three-dimensional finite element model of DLD is proposed for the investigation of the distribution of temperature and residual stress in continuous laser deposition (CLD) and pulsed laser deposition (PLD), which includes continuous pulsed laser deposition (CPLD) and interval pulsed laser deposition (IPLD). This paper aims to utilize the numerical model to analyze the thermal and mechanical distribution characteristics of different deposition patterns. The model enables the accessibility of the deposition process parameters with the integrated activation element strategy, in which the elements of each deposition layer are grouped into one analysis step. The accuracy and stability of the model were verified by experiments. The experiments were carried out with Inconel 718 powder on a three-axis DLD system. Based on the model, the simulation results on the effects of deposition patterns were discussed with the same energy input time of each layer. Considering the research mentioned above, this work can be helpful to reduce the distortion and improve the material properties of complex large parts in the manufacturing department.

## 2. Finite Element Model Methodology

### 2.1. DLD Model

**Figure 1:**
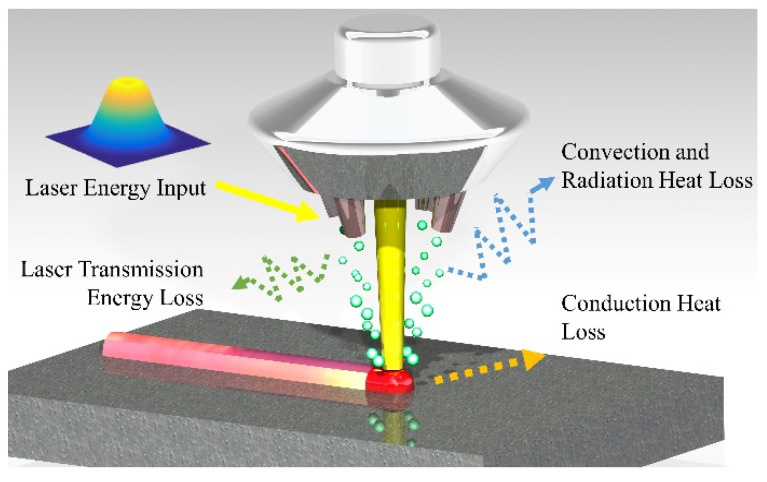
Schematic diagram of the direct laser deposition.

The DLD system consists of the laser equipment, the numerical control workbench, and the powder feeder with four nozzles in symmetric distribution. The metal powder is delivered by argon gas reaching the substrate. The steady powder flow and anti-oxidation circumstances are formed. The DLD process is shown in Figure 1.

The thermal history and mechanical properties of the deposition layer and the substrate are considered in the finite element model (FEM). In order to save the calculation time while ensuring the simulation accuracy, the solidification kinetics and laser–powder coupling influence are simplified. The laser energy is applied on the upper surface of the deposition layer. The solidification process appears after the laser spot departs from the current area. The heat transfer process is composed of heat convection, heat radiation, and heat conduction.

Because this paper aims to study the effects of different deposition patterns on the thermal history and residual stress, the thermophysical parameters are provided for finite element activation and node temperature computation. Then, the temperature data are used in residual stress analyses until all steps are finished. The mesoscopic models used for highly varied and anisotropic characteristics are generally below the millimeter level [23,24]. The macro-model is still the major approach for part-level simulation. In this paper, the general macro-homogeneous material model is applied to the substrate and deposition layer.

### 2.2. Thermal Model of Multi-Layer Deposition

#### 2.2.1. Laser Beam

The proposed DLD model is used to predict the temperature change in the deposition process. The domain temperature is calculated by Equation (1) [14].

(1) $$\rho C_{p}\frac{\partial T}{\partial t}-\nabla \cdot (k_{m}\nabla T)=Q$$

where $\rho \left({\mathrm{kg}/m}^{3}\right)$ is the density of the deposition material; $C_{p}\left(J/\mathrm{kg}/K\right)$ is the specific heat of the deposition material; $T\left(x,\;y,\;z\right)$ is the temperature field in the three dimensional space; *t* is the model computation time; $k_{m}\;\left(W/m/K\right)$ is the thermal conductivity in consideration of the Marangoni effect; and $Q\left({W/m}^{3}\right)$ is the volumetric heat flux density.

The thermal conductivity $k_{m}$ is described in Equation (2), and has been considered to compensate the Marangoni flow effect by modifying thermal conductivity for the FEM model.

(2) $$k_{m}=ck\;T>T_{m}$$

where $k\left(W/m/K\right)$ is the thermal conductivity of the deposition material and *c* is the correction factor, defined as 2.5 [7].

Deposited heat input $q(r)\left({W/m}^{2}\right)$ is described in Equation (3) by a mixture of two fundamental modes with 40% TEM00 and 60% TEM01. The source beam profile is a near flat-top form [25].

(3) $$q(r)=\frac{2\eta P}{\pi r_{0}^{2}}(a+\frac{2br^{2}}{r_{0}^{2}})e^{-2r^{2}/r_{0}^{2}}$$

where a is the fraction of the TEM00 model; b is the fraction of the TEM01 model; $P\left(W\right)$ is the heat source power; $r_{0}(m)$ is the heat source beam radius; and η is the laser absorptivity.

#### 2.2.2. Thermal Model Boundary Conditions

The initial condition of the thermal model in the substrate at time *t* = 0 is described in Equation (4).

(4) $$T_{t=0}=T_{amb}$$

where $T_{amb}$ is the ambient temperature of the substrate.

The convection and radiation boundary condition can be described in Equation (5) [14].

(5) $$\nabla \cdot (k_{s}\nabla T)=h_{c}(T-T_{amb})+\varepsilon_{em}\sigma_{sbc}(T^{4}-T_{amb}^{4})$$

where $h_{c}\left({W/m}^{2}/K\right)$ is the heat convection coefficient; $\varepsilon_{em}$ is the emissivity; and $\sigma_{sbc}\;\left({W/m}^{2}{/K}^{4}\right)$ is the Stefan–Boltzmann constant, defined as 5.67 × 10^−8^.

The latent heat of the deposition melting pool is described in Equation (6).

(6) $$H=\int \rho C_{P}dT$$

where H(J) is the enthalpy.

### 2.3. Mechanical Model of Multi-Layer Deposition

#### 2.3.1. Material Model

The thermal-stress static mechanical analysis is described for the multi-layer DLD model. The stress of each activated layer should be considered in the whole melting and solidification process.

The inertia term and time-dependent term are ignored in the stress analysis model. The stress tensor σ and total strain ε can be described in Equations (7) and (8) [26].

(7) σ=C⋅ε

(8) $$\varepsilon =\varepsilon_{e}+\varepsilon_{p}+\varepsilon_{T}$$

where C is the stiffness tensor; $\varepsilon_{e}$ is the elastic strain; $\varepsilon_{p}$ is the plastic strain; and $\varepsilon_{T}$ is the thermal strain.

The thermal strain is described in Equation (9).

(9) $$\varepsilon_{T}=\alpha (T-T_{ref})$$

where α is the thermal expansion coefficient and $T_{ref}$ is the reference temperature.

#### 2.3.2. Mechanical Model Boundary Conditions

The two short edge fixed constraints are used in the actual DLD process condition in case of the dislocation of the substrate under the rapid movement of the workbench. The nodes on the short edge surface impose the fixed constraint in the FEM.

### 2.4. Model Description

#### 2.4.1. DLD Parameters and Scanning Pattern Illustration

The parameters of the DLD process operation are integrated into the FEM, as in Table 1 [22]. The form of the model was decided by the parameters. The thermophysical parameters chosen are shown in Table 2 and Table 3 [27,28,29,30].

#### 2.4.2. Element Activating Process Illustration

The basic idea of the proposed multi-layer numerical model is to stimulate the temperature field and stress field with the continuous addition of materials according to the moving heat source. In order to ensure the consistency with the DLD process, 20 layers were deposited as a thin wall on the upper surface of the substrate. The elements in each layer were integrated into step *k* (*k* = 1, 2, …). The types of the elements in the FEM were divided into deactivated elements and activated elements. All the elements of the current step were deactivated at the initial stage. The elements that the laser spot scanned through were activated after each analysis step. The element activation sequence was implemented with the predetermined motion path of the NC program. The analysis step was terminated when all elements were activated.

The element activation criteria can be described in Equation (10).

(10) $$\left\{\begin{array}{c} x_{mean(i)}=(x_{mean(i-1)}+x_{coord(i)})/N_{node(i)}\\ y_{mean(i)}=(y_{mean(i-1)}+y_{coord(i)})/N_{node(i)}\\ {\left(x_{t}-x_{mean(i)}\right)}^{2}+{\left(y_{t}-y_{mean(i)}\right)}^{2}\leq r_{0}^{2} \end{array}\right.$$

where $x_{mean(i-1)}$ is the mean value of *x* direction node coordinates before activation; $y_{mean(i-1)}$ is the mean value of *y* direction node coordinates before activation; $x_{mean(i)}$ is the mean value of *x* direction node coordinates after activation; $y_{mean(i)}$ is the mean value of *y* direction node coordinates after activation; $x_{coord\left(i\right)}$ is the *x* coordinate activation value of step *i*; $y_{coord\left(i\right)}$ is the *y* coordinate activation value of step *i*; $N_{node\left(i\right)}$ is the coordinate points sum of step *i*; $x_{t}$ is the simulation coordinate value of *x* direction; and $y_{t}$ is the simulation coordinate value of the *y* direction.

**Figure 2:**
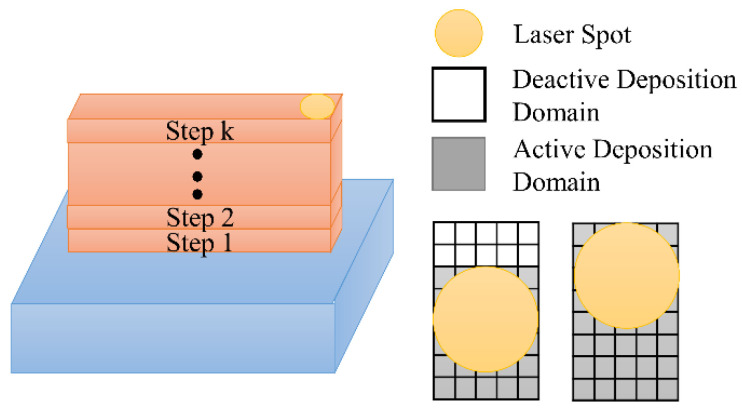
Diagram of element activation scheme.

The elements were activated in the simulation process, as shown in Figure 2. The activation method is continuous for metal powder deposition in the time scale. All elements of the layers were regarded as homogeneous blocks in consideration of the simplified calculation, which ignored the arc region due to molten metal flow.

#### 2.4.3. DLD Simulation Model Architecture

**Figure 3:**
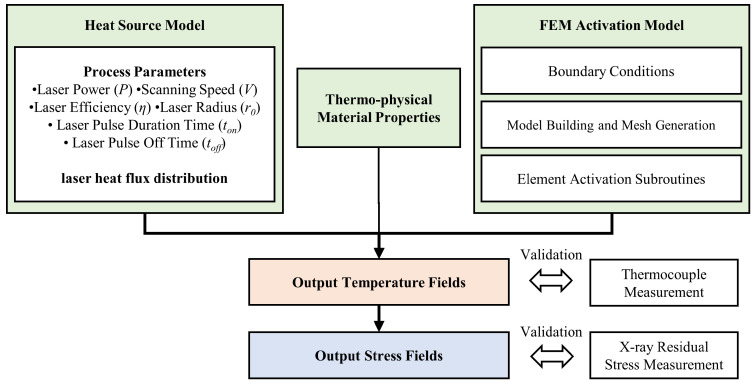
Flowchart of numerical simulation for temperature and stress prediction.

The DLD simulation model consists of the heat source model, the thermophysical material properties, and the FEM activation model, as shown in Figure 3. The laser pulse duration $t_{on}$ and the laser pulse off time $t_{off}$ were introduced into the heat source model for the CPLD process and IPLD process. The thermophysical material properties were considered for the transient heating and cooling process of the metal deposition. The temperature field obtained by the model is used for thermal stress calculation. The thermophysical properties of Inconel 718 are depicted in Table 2.

**Figure 4:**
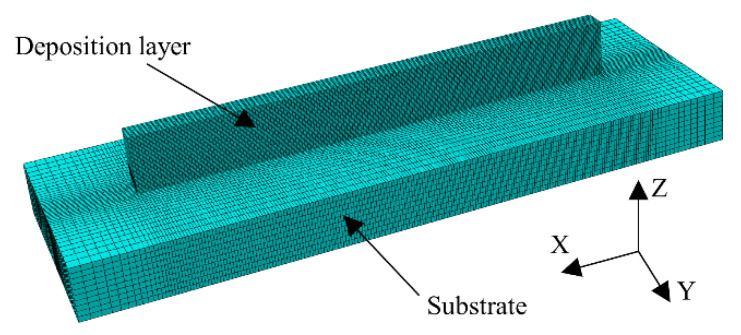
Meshed finite element model.

Based on the above architecture, ABAQUS and PyCharm software were used for the model building and the subroutine development. Model building considered the size of the substrate and the deposition layers. The finest mesh element was used in the deposition area and the area adjacent to the substrate, as shown in Figure 4. The minimum size of the mesh element was 0.13 mm. The total number of the mesh elements was 73,600. The heat transfer step and the static general step were used for the analyses of the temperature fields and the stress fields. The convective heat transfer boundary condition and the thermal emissivity boundary condition were imposed on the external mesh surface. The predefined field temperature was set to the ambient temperature. The thermocouple measurement and X-ray diffraction (XRD) residual stress measurement were used for the simulation data validation.

## 3. Validation

To validate the numerical model, the deposition substrate was embedded by three groups of thermocouples for the acquisition of thermal fields. The temperature logger was used to store temperature data. The XRD instrument was used for the validation of the substrate residual stress.

### 3.1. DLD Experimental Setup

**Figure 5:**
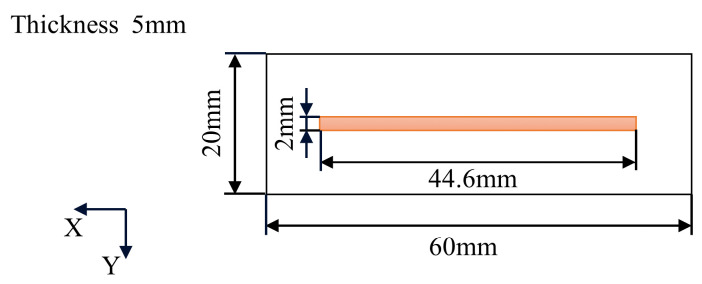
Diagram of deposition area and substrate dimensions.

**Figure 6:**
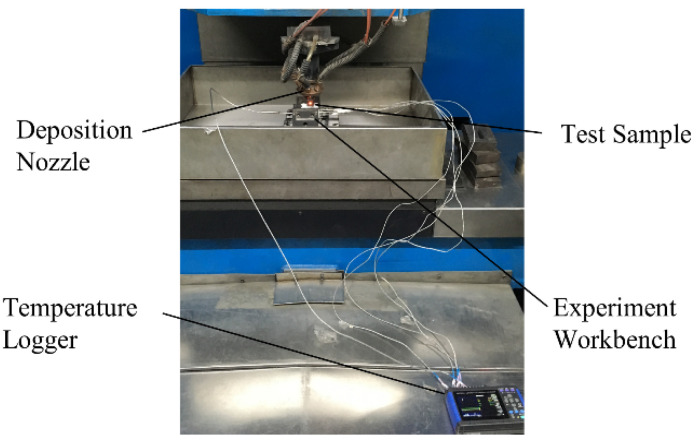
Experimental setup and temperature measuring equipment.

Inconel 718 powders with a size distribution of 45–105 μm were used in the experiment. For consistency of experimental conditions, the Inconel 718 cold-rolled plate was used with a size of 60 mm × 20 mm × 5 mm, as shown in Figure 5. A 3 kW CO_2_ laser with a 2 mm spot diameter was used in the DLD experiment system. The CNC system was used for motion control of the laser head and powder feeding nozzle in the three-axis working platform, as shown in Figure 6. The working condition of argon shielding gas was 10 L/min flow rate and 0.1 MPa pressure, which was used to protect the deposition layer from oxidation.

**Figure 7:**
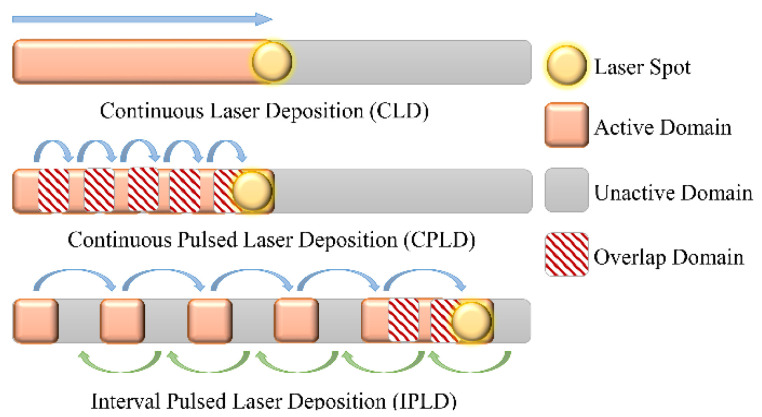
Representative three deposition patterns.

The movement patterns of the three-process plan are shown in Figure 7. The laser power of the three-process plan was 1800 W. The laser scanning speed of the continuous laser deposition (CLD) was 5 mm/s. The laser pulse duration of the continuous pulsed laser deposition (CPLD) and the interval pulsed laser deposition (IPLD) was 0.25 s for the consistency of the laser input energy of each layer. Based on these considerations, the total deposition time of the CLD was 200 s, and the total deposition time of the CPLD and IPLD was 365 s. In order to ensure the quality of the deposition layer, the overlap rate of the CPLD process and IPLD process was set to 33%. The number of deposition points in each layer was 33.

### 3.2. Temperature Measurement

**Figure 8:**
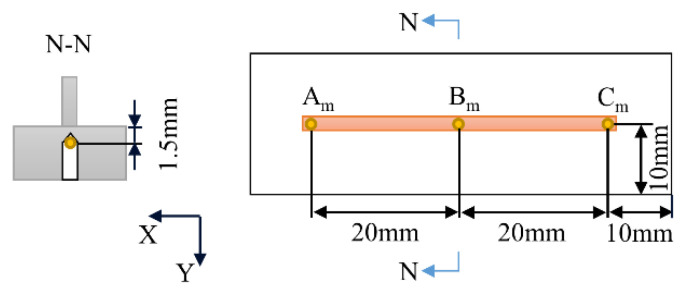
The location of temperature measuring point.

HIOKI-LR8431 temperature logger and K-type thermocouples were used for the temperature measurement. The sampling frequency of temperature data was 10 ms. Three test holes were drilled in the substrate for inserting the probe thermocouples. The diameter of the test holes was 3 mm, as shown in Figure 8. The top of the test holes was 1.5 mm away from the upper surface of the substrate. The spacing between holes was 20 mm and arranged symmetrically.

### 3.3. Residual Stress Measurement

**Figure 9:**
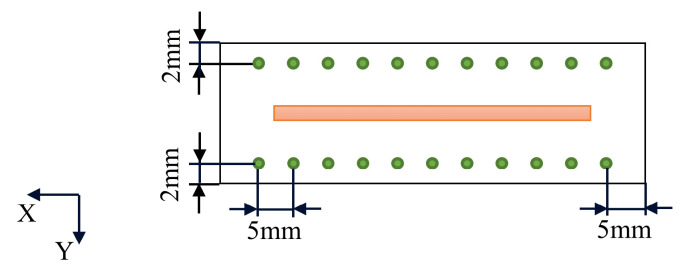
Location of temperature measuring point.

The Pulstec μ-X360s XRD instrument was used for deposited substrate residual stress measuring. The change in atomic plane distance and the influence on diffraction peak displacement were measured by X-ray. The corresponding residual stress values were obtained by comparing with the standard sample. The X-ray diffraction angle θ and the wavelength λ were used for the calculation of the atomic plane distance in Bragg’s law. The 2θ angle used in the software was 151°. Two groups of measurement points parallel to the deposition direction were selected. As shown in Figure 9, the distance between the measured points was 5 mm. The stress results obtained in the experiment were used to compare with the simulation results.

## 4. Results and Discussion

### 4.1. Thermal Results

**Figure 10:**
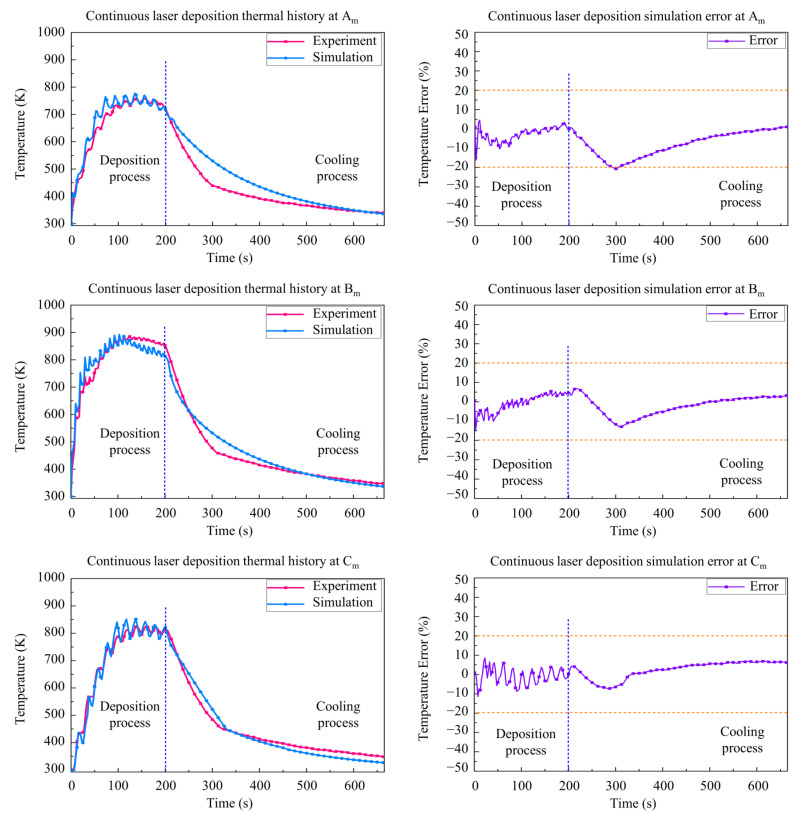
Comparison between simulation and experimental results for continuous laser deposition.

As shown in Figure 10, the simulation results and experimental results of three measuring points—$A_{m}$, $B_{m}$*,* and $C_{m}$ are given to validate the accuracy of the CLD process. It can be seen that the simulation temperature results were in good agreement with the experimental temperature results. The maximum temperature of the CLD process is about 900 K in the deposition stage. Due to the existence of heat accumulation, the temperature at $B_{m}$ is higher than the value at $A_{m}$ and $C_{m}$. The cooling rate decreased gradually after a cooling process of about 100 s.

Due to the rapid heating and cooling, relatively higher differences are found in the initial stage and the beginning of cooling stage. The difference between the simulation and experiment of the CLD processes is below 20% in most of the process time, which indicates the accuracy of the numerical model. In the initial cooling stage, the temperature changes dramatically, which leads to the strong convection of shielding gas near the deposition layer. The maximum error is caused by the change of heat transfer environment. It is found that the error increases during the beginning and end of deposition processes. One of the reasons is the error of the thermocouple and data recorder when the temperature changes suddenly. The change in protective atmosphere at the beginning and end of the deposition process should be the other reason that changes the heat convection of the deposition layer and the substrate. Similar error results have been recorded in relevant studies [3,31], which had acceptable stress simulation results. In order to obtain more accurate temperature results in future research, shields should be established to protect the thermocouples from the effect of convection. Equipment and control procedures should be improved to provide a stable atmosphere at the beginning and end of the deposition process.

**Figure 11:**
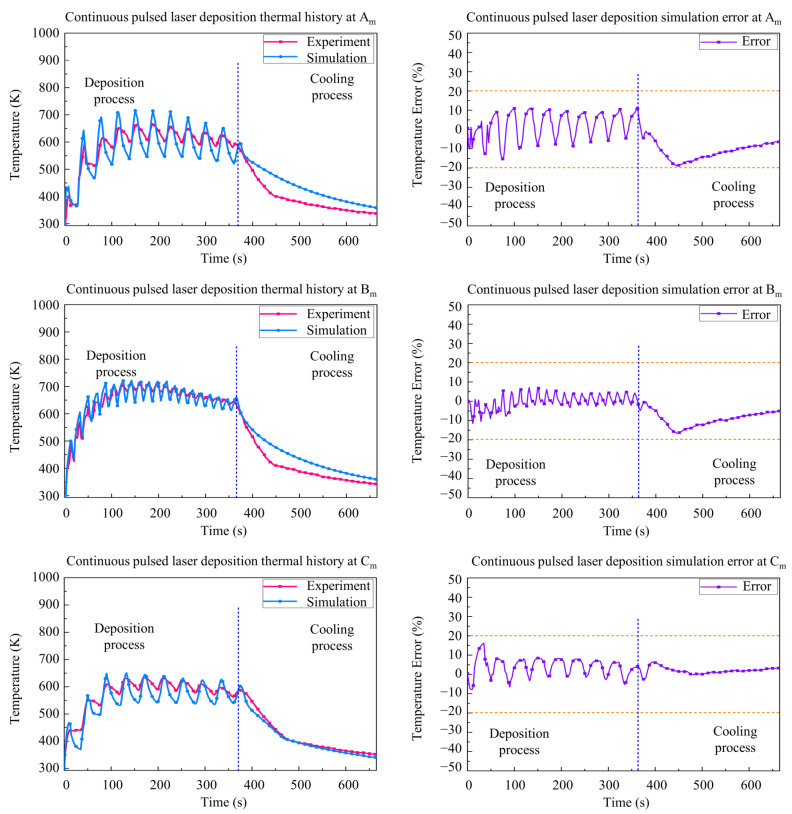
Comparison between simulation and experimental results for continuous pulsed laser deposition.

**Figure 12:**
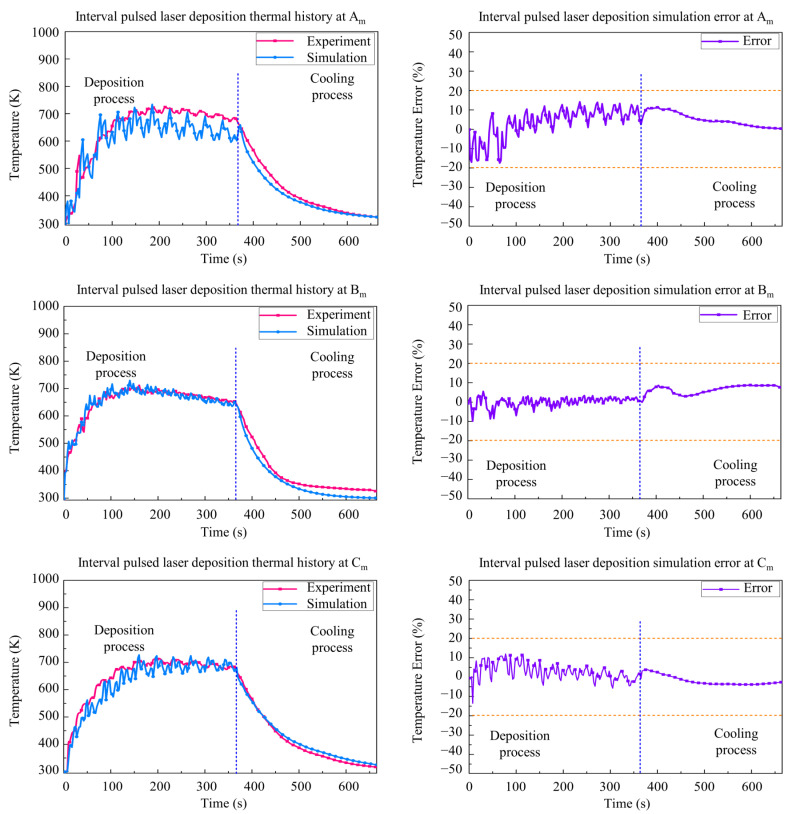
Comparison between simulation and experimental results for interval pulsed laser deposition.

As shown in Figure 11 and Figure 12, the simulation results and experimental results of the three measuring points $A_{m}$, $B_{m}$, and $C_{m}$ are given to validate the accuracy of the CPLD process and the IPLD process. It can be seen that the simulation temperature results are also in good agreement with the experimental temperature. The maximum temperature is about 700 K in the deposition stage. The maximum temperature is found at $B_{m}$, but the measuring temperature results are lower than the CLD process. Large temperature fluctuation is found at the edges of the deposition layer due to the pulse interval time in the CPLD process. However, the temperature fluctuation of the IPLD process is lower than that of the CPLD process, because of a reciprocating deposition route at each layer.

A relatively higher difference between the simulation and experiment is found in the initial stage and the beginning of cooling stage. The difference values of the CPLD process and the IPLD process are lower than that of the CLD process. The results fluctuated in the deposition process due to the periodic pulsed deposition.

### 4.2. Residual Stress Results

**Figure 13:**
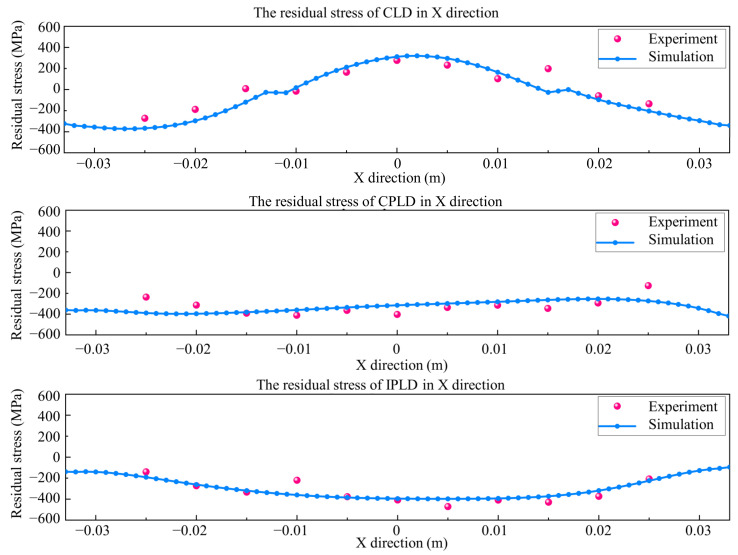
Residual stress comparison between simulation and experimental results.

In this section, the validation of residual stress is conducted in terms of measurement point data. The comparison of residual stress between the simulation data and experiment data is shown in Figure 13. The simulation results of the three processes are in good agreement with the experimental results. The maximum error is shown between the fixed end and the end of the deposition domain. The error would have been caused by the clamping constraints and the intense temperature change at the edge of the deposition domain. The measured residual stress data are between −400 MPa and 400 MPa. The transformation of tensile and compressive residual stress is also consistent with the distortion of substrate. The experimental results of residual stress are similar to previous research results. The measured compressive stress data are affected by the substrate machining stress and the constraints during deposition. It was found that the change trend in residual stress between the simulation and experiment was consistent, which indicates the accuracy of numerical model.

### 4.3. Discussion

**Figure 14:**
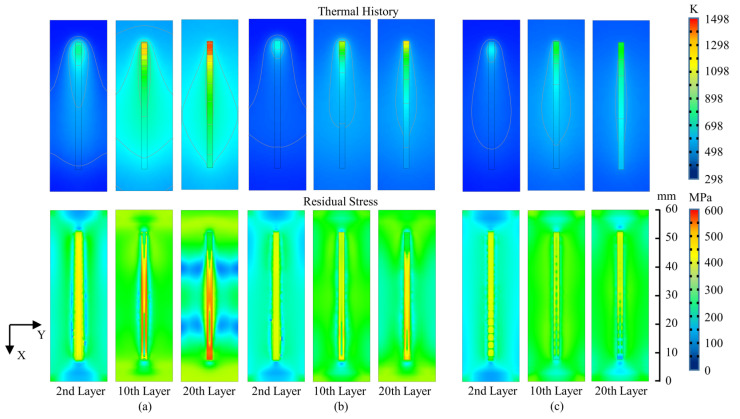
Comparison of three patterns of thermal history (K) and residual stress (von Mises stress, MPa) in deposition processes: (**a**) CLD; (**b**) CPLD; (**c**) IPLD.

In order to study the regulation of thermal history and residual stress in the deposition process, three groups of data graphs selected from bottom to top are shown in Figure 14. After the deposition of the second layer, it can be seen that heat concentration appears in the end region of the CLD process. The temperature of the whole substrate increases significantly when the 10th layer deposition is finished. The highest temperature in the end region of CLD process is close to 1400 K when the last layer is deposited, while the temperature of pulsed deposition processes is about 900 K.

It can be seen that the change in residual stress is not obvious after the deposition of the second layer, and larger residual stress can be observed in the sides of the substrate. After the deposition of the tenth layer, the stress concentration is found near the deposition layer of the CLD process. After the deposition of the 20th layer, it can be seen that the stress distribution of the CLD process tends to be evidently inhomogeneous. Better homogeneity is shown in the stress distribution of the IPLD process.

**Figure 15:**
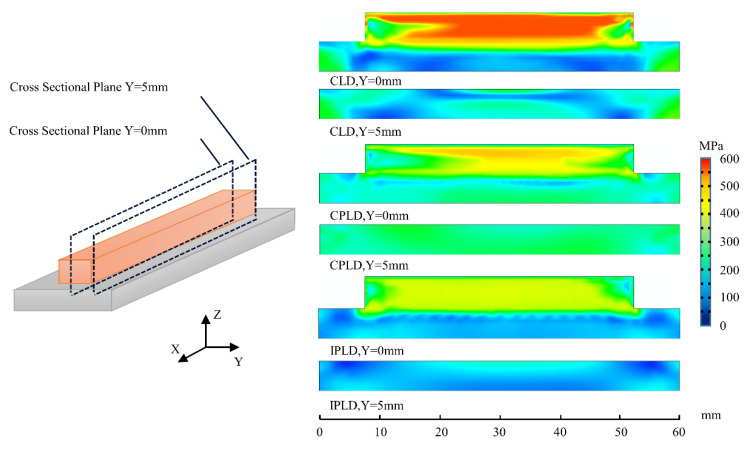
Comparison of three patterns of residual stress at cross-section plane (von Mises stress, MPa).

Figure 15 depicts the residual stress distribution of the longitudinal section after the whole deposition process is finished. It is obvious that the stress in the deposition layer is not homogeneously concentrated in the CLD process, and the high stress area is more concentrated in the middle. The stress concentration of the CPLD process is lower than the CLD process, but there is still some uneven distribution. The peak value of residual stress in the IPLD process is the lowest of the three processes, and the distribution is more homogeneous in the whole deposition layer.

**Figure 16:**
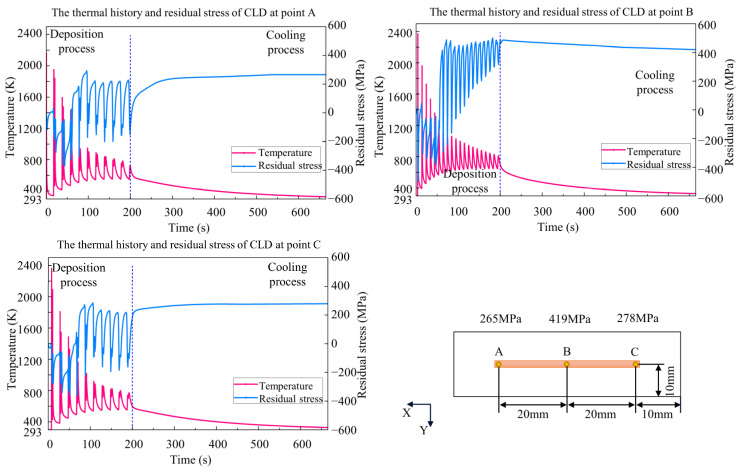
Comparison of thermal history and residual stress *S_xx_* value in CLD.

**Figure 17:**
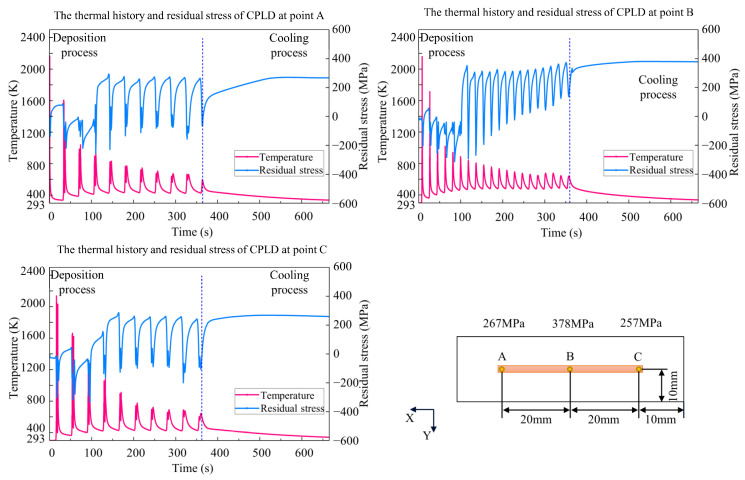
Comparison of thermal history and residual stress *S_xx_* value in CPLD.

**Figure 18:**
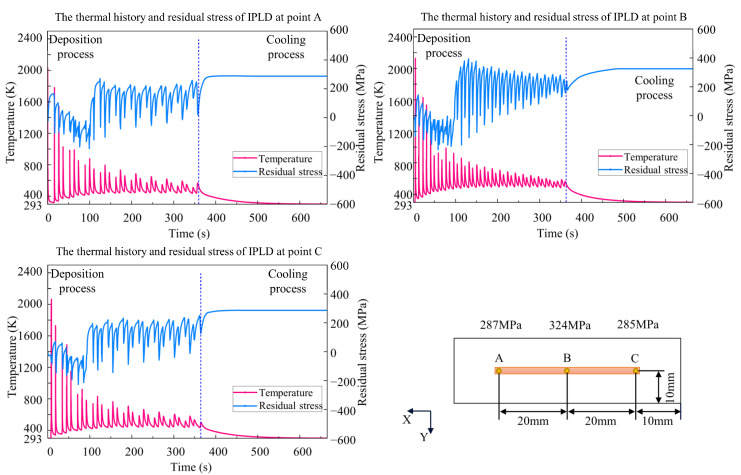
Comparison of thermal history and residual stress *S_xx_* value in IPLD.

Because this paper aimed to study the effects of thermal history on residual stress, the adjacent domain between the deposition layer and the substrate is a good observation target. Three points, A, B, and C, at the bottom of the deposition layer were selected to compare temperature and residual stress along the x direction. During the deposition process, the temperature-fluctuation range of the three processes becomes lower, while the residual stress increases with repeated heating and cooling. As shown in Figure 16, Figure 17 and Figure 18, the temperature fluctuates widely at the initial stage. As the molten pool area gradually moves away from the measuring point, the maximum temperature and the fluctuation amplitude gradually decreases. The temperature at center point B of the CLD process is the highest, significantly higher than that at points A and C. The fluctuation frequency of the IPLD process is higher than that of the CLD and CPLD processes because there is a round-trip movement in each layer. The temperature results of the CPLD and IPLD processes are more homogeneous at the three measuring points. Because melting and solidification occur at the bottom interface at the initial stage, the amplitude of stress fluctuation is smaller. When the molten pool moves away from the measuring point, the bottom interface endures the solidification state. Repeated expansion and contraction of the bottom interface leads to greater stress fluctuation. The fluctuation amplitude decreases and remains stable until the heating area is far enough away from the bottom. Due to the short deposition process and obvious heat concentration, the residual stress at point B in the CLD process is significantly higher than that in the CPLD and IPLD processes. The residual stress difference between the edge position and the center position of the IPLD process is low.

**Figure 19:**
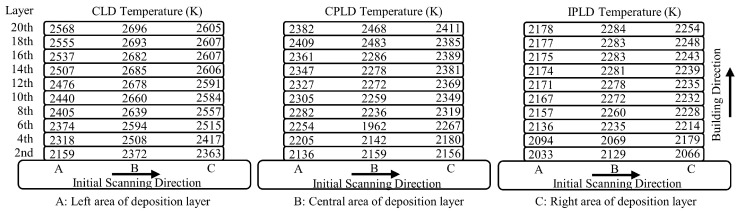
Simulated temperature results at the end of every two layers.

In order to depict the temperature changes in the building direction, the temperature results at the start and end points of every two deposition layers are shown in Figure 19. It can be seen that the temperature of side A is low at the beginning. With the increase in height of the deposition layer, the middle temperature is higher than the edge temperature. Due to the effect of laser pulse off time, the maximum temperature of the CLD process is about 200 K higher than that of the CPLD process. IPLD process temperature increases slowly with the increase in building height. The temperature difference of the IPLD process between the middle and both sides of the deposition layer is the smallest among the three processes.

**Figure 20:**
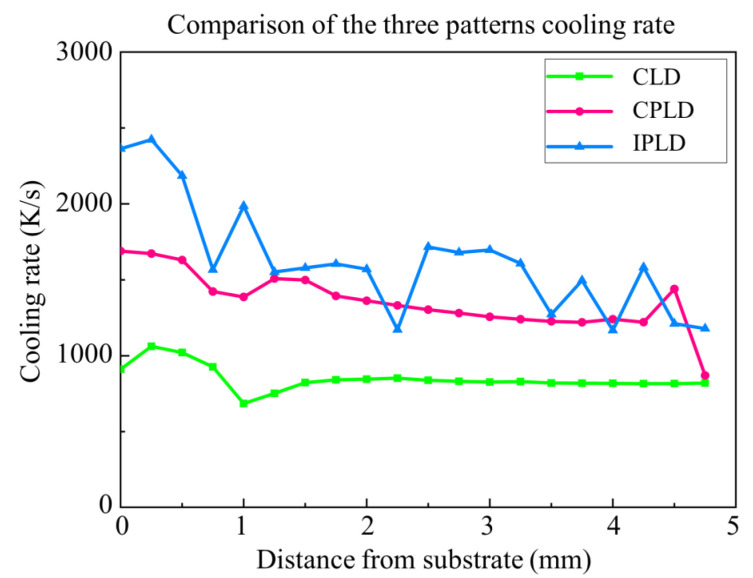
Comparison of the three patterns’ cooling rate.

The heat concentration condition is also reflected in the temperature-change rate in the building direction. The cooling rate results of the deposition layers of the three processes are shown in Figure 20. The initial cooling rate of the IPLD process is the fastest. The initial cooling rate of the CLD process is the lowest, due to lack of laser off time and the decreasing of temperature difference between the substrate and the deposition layer. With the increase in distance from the substrate, the cooling rates of the three processes decrease gradually. A similar cooling rate downward can also be found in the literature [32]. Due to heat accumulation, the cooling rate decreased rapidly until it is close to 1 mm. When the molten pool area is completely away from the substrate, the side surfaces of the deposition layer are rapidly cooled by the argon flow, resulting in the transient increase in the cooling rate. After a short period of fluctuation upward, the cooling rate decreases slowly due to the continuous heat accumulation in the deposition layer.

In this paper, the error between the prediction of temperature and stress and the experimental results is below 20%, which is equivalent to the results of related studies [3,33]. In the model, the DLD simulation is realized for CLD, CPLD, and IPLD processes with multi-layer continuous material addition. The simulation method can also be applied to the temperature and stress prediction of the deposition process of other complex path components. The solid phase transition of the substrate and the deposited layer is ignored in this paper, but the temperature and stress changes caused by material phase transition at the interface is still a worthy research direction. Fang et al. [34] simulated the phase transition with three deposition layers, but the accurate measurement of phase properties and the computational efficiency are still challenging.

Among the three main heat transfer modes (conduction, convection, and radiation), heat conduction accounts for more than 90% [1]. It can be seen from the simulation results that the change in heat conduction state caused by the discrete heat input can significantly affect the heat distribution and cooling process in the deposited parts. As shown in Table 4, the maximum central temperature and maximum residual stress of the IPLD process are the lowest. The IPLD process is considered to obtain more homogeneous distribution of temperature fields and stress fields. However, the deposition time of the CPLD and IPLD processes increased by 82.5%.

## 5. Conclusions

In this paper, a multi-layer deposition numerical model was proposed to predict the temperature field and residual field with the finite element method. Unlike most previous studies, the novelty of the proposed model is to simulate the deposition process of CLD and PLD processes by controlling the same laser input time of each layer. Conclusions and findings include:

1. The temperature results obtained from the proposed model show reasonable agreement with the experimental data collected from thermocouples (results of three measuring points show errors within 10%, while the maximum error at the beginning and end of the experiment is close to 20%).
2. At the end of each layer, the temperature fields show that two PLD processes cool faster than the CLD process. This indicates that the pulse interval reduces the accumulation of heat in the deposition layers.
3. The proposed IPLD process shows more homogeneous residual stress distribution, which is attributed to lower intensity changes in the temperature field and the thermal stress effect.
4. With the increase in the number of layers, the maximum temperature of the three processes increases gradually, and the CLD process temperature shows the fastest growth. Due to heat conduction, the temperature in the middle area of each layer is higher than that on both sides. At the beginning of deposition, the cooling rate of the IPLD process is the highest, but the values of the three processes decrease gradually with the deposition process.

The reported findings could be meaningful to provide a reference for controlled heat accumulation and stress concentration in the DLD process. the IPLD process is suitable for thin-walled parts that are greatly affected by distortion. For larger parts, the efficiency of the deposition process still has to be considered.

## Figures and Tables

**Table 1:** Deposition process parameters.

Deposition Parameters	Value
Laser power *P*	1800 W
Laser scanning speed *v*	0.005 m/s
Laser turn-on time *t_on_*	0.25 s
Laser turn-off time *t_off_*	0.25 s
Laser absorptivity *η*	0.12
Laser beam radius *r*	0.001 m
Powder feeding rate	10 g/min
Powder type	Inconel 718
Initial temperature	298 K

**Table 2:** Thermal properties of Inconel 718.

Temperature (K)	Density (kg/m^3^)	Emissivity	Thermal Conductivity (W/mK)	Specific Heat Capacity (J/kgK)	Thermal Linear Expansion $\left(m/m/{}^{\circ}C\;\times \;10^{-6}\right)$
298	8190	0.539	8.9	435	12.8
373	8160	0.533	10.8	455	13.2
473	8118	0.533	12.9	479	13.6
573	8079	0.534	15.2	497	13.9
673	8040	0.534	17.4	515	14.1
773	8001	0.535	18.7	527	14.4
873	7962	0.535	20.8	558	14.9
973	7925	0.536	21.9	568	15.5
1073	7884	0.536	26.9	680	16.3
1173	7845	0.537	25.8	640	17.5
1273	7806	0.537	26.7	620	19.2
1373	7767	0.538	28.3	640	21.9

**Table 3:** Mechanical properties of Inconel 718.

Temperature (K)	Modulus of Elasticity (GPa)	Poisson’s Ratio	Yield Stress (MPa)
294	208	0.3	1117
366	205	0.3	1117
477	202	0.3	1112
589	194	0.3	1110
700	186	0.3	1080
811	179	0.3	1070
922	172	0.3	1030
1033	162	0.3	758
1144	127	0.3	592
1227	17.8	0.3	468
1533	2.08	0.3	11.7

**Table 4:** Comparison of three process differences.

Process Classifier	Maximum Temperature (K)	Temperature Difference between Center and Edge (K)	Maximum Bottom Residual Stress (MPa)	Residual Stress Difference between Center and Edge (MPa)	Deposition Time (s)
CLD	2696	109.5	419	147.5	200
CPLD	2468	71.5	378	116	365
IPLD	2284	68	324	38	365
